# Genotypic Analysis of Kaposi’s Sarcoma-Associated Herpesvirus from Patients with Kaposi’s Sarcoma in Xinjiang, China

**DOI:** 10.3390/v6124800

**Published:** 2014-11-26

**Authors:** Xinxing Ouyang, Yan Zeng, Bishi Fu, Xiaowu Wang, Wei Chen, Yuan Fang, Minhua Luo, Linding Wang

**Affiliations:** 1 State Key Laboratory of Virology, Wuhan Institute of Virology, Chinese Academy of Sciences, Wuhan 430071, China; E-Mails: annlooy@gmail.com (X.O.); fubishi001@yeah.net (B.F.); 2 Laboratory of Xinjiang Endemic and Ethnic Diseases, Shihezi University, Shihezi 832002, China; E-Mail: yzeng910@163.com; 3 Department of Microbiology, Anhui Medical University, Hefei 230032, China; E-Mails: Wangxiaowuxiaowu@sina.com (X.W.); chenwei1413@163.com (W.C.); yuanslience@163.com (Y.F.); 4 Central Laboratory of Molecular and Cellular Biology, School of Basic Medicine, Anhui Medical University, Hefei, Anhui 230032, China

**Keywords:** Kaposi’s sarcoma-associated herpesvirus (KSHV), genotyping, K1 gene, Xinjiang

## Abstract

Kaposi’s sarcoma-associated herpesvirus (KSHV) is the causal agent of all forms of Kaposi’s sarcoma (KS), including AIDS-KS, endemic KS, classic KS and iatrogenic KS. Based on Open reading frame (ORF) K1 sequence analysis, KSHV has been classified into seven major molecular subtypes (A, B, C, D, E, F and Z). The distribution of KSHV strains varies according to geography and ethnicity. Xinjiang is a unique region where the seroprevalence of KSHV is significantly higher than other parts of China. The genotyping of KSHV strains in this region has not been thoroughly studied. The present study aimed to evaluate the frequency of KSHV genotypes isolated from KS tissues in Classical KS and AIDS KS patients from Xinjiang, China. ORF-K1 of KSHV from tissue samples of 28 KS patients was amplified and sequenced. Two subtypes of KSHV were identified according to K1 genotyping. Twenty-three of them belonged to subtype A, while five of them were subtype C. More genotype A than genotype C strains were found in both Classical KS and AIDS KS. No significant difference was found in the prevalence of different genotype between Classical KS and AIDS KS.

## 1. Introduction

Kaposi’s sarcoma (KS) is a tumor, originally described by Moritz Kaposi in 1872 [1] and became more widely known as one of the AIDS defining illness in the 1980s [2]. The clincoepidemiologic forms of KS have been classified as AIDS-KS, classic KS, endemic KS and iatrogenic KS [3]. Classic KS was an indolent cutaneous disease entity primarily involving the lower extremities, affecting elderly men especially from the Mediterranean region or of Eastern European decent [4,5]. In contrast, AIDS-associated KS is clinically more aggressive and can occur in all organs with the exception of the central nervous system; it stimulated the greatest interest as one of the first illness associated with AIDS [6,7].

Kaposi’s sarcoma-associated herpesvirus (KSHV), also known as human herpesvirus 8 (HHV-8), is a gamma-2-herpesvirus or rhadinovirus. KSHV was first identified in a biopsy tissue from a patient with AIDS-related Kaposi’s sarcoma (AIDS-KS) by representational difference analysis in 1994 by Chang *et al.* [8]. KSHV is the causal agent of all forms of Kaposi’s sarcoma, including AIDS-KS, endemic KS and iatrogenic KS (in transplant recipients receiving immunosuppressive therapy) [8]. KSHV is also associated with two other lymphoproliferative malignancies, including primarily effusion lymphoma and multicentric Castleman’s disease (MCD) [9,10].

As a large double-stranded DNA virus, KSHV has approximately 90 identified open reading frames, of which over 60 show homology with other rhadinoviruses and 15, designated K1–K15, were unique to KSHV when its genome was first sequenced [11]. ORF-K1, at the left end of KSHV genome, encodes an early lytic transmembrane glycoprotein of 289 amino acids (aa). The amino acid sequence of K1 varies from 0.4% to 44% between different KSHV isolates, with the variations concentrated in two hyper-variable regions, VR1 and VR2. Current genotyping method of KSHV is based primarily on the sequence variations of the ORF-K1 gene. Based on K1 sequence analysis, KSHV has been classified into seven major molecular subtypes (A, B, C, D, E, F and Z) [12,13,14,15,16,17]. The distribution of KSHV strains varies according to geography and ethnicity, which appears to be attributable to human migrations. Subtype A and C are found in Europe, the USA, Middle East and Northern Asia; Subtype B is characteristic for Africa; Subtype D was found in individuals from the pacific Islands; Subtype E was found in Brazilian Amerindians; Subtype Z has been found in a small cohort of Zambian children; A new subtype F has been recently identified in Uganda.

Xinjiang Uygur Autonomous region is the largest province in northwestern China, and located on the ancient Silk Road as an important staging post over a thousand years ago. Xinjiang borders on Russia, Kazakhstan, Kyrgyzstan, Tajikistan, Pakistan, Mongolia, India and Afghanistan. Ethnic groups in Xinjiang are diverse and distinct, the main ethnic groups are the Uygur (45.7%) and the Han (39.7%), other ethnic minorities include Kazakh, Mongolians, Hui, Kirgiz, Manchu, and Xibo. Classic KS are rarely seen in the Han Chinese, but are seen more frequently in the Uygur ethnic group, more than one patient has been diagnosed histopathologically as having KS every year at the Affiliated Tumor Hospital of Xinjiang Medical University in Urumqi, the capital city of Xinjiang Uygur Autonomous Region. We have previously shown that the overall seroprevalence of KSHV was 19.2% in the general population in Xinjiang, which was substantially higher than the 9.5% seroprevalence of KSHV in the control subjects from the general population in Han Chinese in Hubei Province [18]. Our data indicated that Xinjiang is a unique region where the seroprevalence of KSHV is significantly higher than other parts of China. This high seroprevalence of KSHV is consistent with the high incidence of KS in this region.

Dilnur *et al.* (2001) had reported that KSHV strains from seven patients with classical KS in Xinjiang were classified as subtype C [19]. Zhang reported that, on the basis of the K1/VR1 amino acid sequence, that the majority of these KS patients were infected by subtype C (*n* = 18), and several by subtype A (*n* = 4) [20]. In this study, we collected 28 samples of KS patients in Xinjiang and examined the infection of KSHV by nested PCR and characterized the ORF-K1 genotypes.

## 2. Materials and Methods

### 2.1. Tissue Specimens

Twenty-eight KS classical tissue examples were collected from Xinjiang Uygur Autonomous Region, China. Seven of them were peripheral blood mononuclear cells examples and the rest were formalin-fixed paraffin-embedded tissues (FFPET). All the specimens were collected between 1997 and 2009. Eighteen of them were from Uygur patients, eight of them were from Kazakh Patients, one from a Hui patient and one from a Han patient. Permission to conduct the study and informed consent was obtained in accordance with a protocol approved by the Ethics Committee of Wuhan Institute of Virology, Chinese Academy of Sciences.

### 2.2. Preparation of DNA

DNA from peripheral blood mononuclear cells of KS patients was extracted with a Blood & Cell Culture DNA Mini Kit according to manufacturer’s instructions (Qiagen, Valencia, CA, USA).

DNA from FFPET was extracted as the following steps. Tissues were incubated at 55 °C with 1 mL xylene for 10 min, and then centrifuged at 8000 rpm for 5 min, supernatant was collected. The supernatant was further centrifuged at 8000 rpm for 5 min to totally remove the paraffin. The remaining dimethylbenzene was removed by using anhydrous ethanol. Then the supernatant was added with 250 μL cell lysis buffer (0.02 M Tris-HCl pH 8.0, 0.01 M EDTA pH 8.0, 2% SDS and 50 μL proteinase K (20 mg/mL) and kept at 55 °C over night. Equal volumes of phenol, chloroform, and isoamyl alcohol (25:24:1) were added to the supernatant. The supernatant was clarified by centrifugation at 8000 rpm for 2 min at 4 °C. Then 2.5 times the volume of pre-cold anhydrous alcohol was added to precipitate DNA. 30 min later, the mixture was centrifuged at 13,000 rpm for 10 min at 4 °C. The supernatant was discarded. After slightly drying, 20 μL TE (pH8.0) was added to dissolve DNA, and kept in −20 °C.

### 2.3. PCR Amplification

Fragments (363 bp) of the VR1 region of KSHV ORF-K1 were amplified by nested-PCR. Primers were designed as described [16]. Briefly, the forward and reverse primers for first round of PCR were 5’-GACCTTGTTGGACATCCTGTA-3’ and 5’-GAGTTTCTGGAGTTATATTG-3’. Primers for the second round of PCR were 5’-TTGTGCCCTGGAGTGATT-3’ and 5’-CAGCGTAAAATTATAGTA-3’. The PCR program was set as 35 cycles 95 °C for 1 min, 53 °C for 45 s, and 72 °C for 1 min in the first reaction; and 94 °C for 1 min, 48 °C for 45 s, and 72 °C for 1 min in the second reaction. PCR products were evaluated in 1.5% agarose gel electrophoresis and stained with ethidium bromide. Afterward, the products were purified using a Gel Extraction Kit (Omega Bio-Tek, Winooski, VT, USA) according to instructions of manufacturer.

### 2.4. DNA Sequencing

The purified K1 gene fragments were cloned into pGEM-T vector for constructing recombinant plasmid pGEM-T-K1 and transformed into DH5α competent cells. Then, the recombinant plasmid was extracted from DH5α cells using TIANprep Mini Plasmid Kit (TIANGEN BIOTECH CO., LTD, Beijing, China) and sent for sequencing by Shanghai Majorbio Biotech Co., Ltd. (Shanghai, China)

### 2.5. Phylogenetic Tree Analysis

Twenty-eight of the unique sequences (GenBank Accession numbers: KM582680–KM582707) and 19 other KSHV strains obtained from GenBank were used to construct the phylogenetic tree and were analyzed with a DNAStar package and aligned with Clustal W in Bioedit (version 7.0.0). Phylogenetic trees were constructed by neighbor-joining analysis by Phylip (version 3.68) and MEGA (version 4.0.2), respectively. The statistical reliability of the NJ tree was evaluated using 1000 bootstrap samples. TreeView (version 1.6.6) was used to see the trees constructed by Phylip. The sequences of 19 other KSHV strains consisted of 1 strain of subtype A1 XJ-27 (FJ853386); 1 strain of subtype A2: Ema-7 (AF130305); 1 strain of subtype A3: IT-268 (GU097421), 1 strain of subtype A4: BCBL-B (AF133039),US114 (GU097431) ; 1 strain of subtype A5: KE229 (GU097433); 3 strains of subtype B: UG-65 (FJ884618), UG-85 (FJ884620), MP10 (AF387367); 2 strains of subtype C2: XJ-6 (FJ853368), XJ30 (FJ853388); 1 strain of subtype C3: XJ-20 (FJ853379), AF17170531; 2 strains of subtype D: TKS10 (AF133043), ZKS3 (AF133044), 2 strains of subtype E: Sio1 (AY329025), Tupi1 (AF220292); and 2 strains of subtype F: AF178810, FJ884616.

### 2.6. Statistical Analysis

All the information of cases was analyzed by descriptive statistics. MS Excel was used to calculate the mean, standard deviation, coefficient of variation, and median of data. Fisher's exact test was used to test the associations of sex ratio and KSHV genotype between two groups and the associations of KS subtypes and KSHV subtypes. One-way analysis of variance (ANOVA) was used for statistical evaluation of differences between the two groups.

## 3. Results

### 3.1. KS Cases Characterization

Twenty-eight KS cases were evaluated for ORF-K1 genotype analysis. The number of male patients was higher than that of female patients. Male patients were 24/28 (85.71%), while female patients were 4/28 (14.29%). Classic KS cases were twenty-three, while AIDS-associated KS were five. Classic KS has a male/female ratio of 6.67 (20/3), and AIDS-associated KS has Male/Female Ratio of 4.0 (4/1). Compared to AIDS KS, the male/female ratio in Classic KS is higher but without significant difference by Fisher’s exact test. The mean ages for Classic KS and AIDS-KS were 54.39 and 23.80 years old, respectively. AIDS associated KS patients were younger than Classic KS (*p* < 0.001; ANOVA). (Table 1).

**Table 1:** Sex and age for KS patients studied.

KS Groups	M/F Ratio	Age						
		Mean (Years)	SD (Years)	CV (%)	P_25_	P_75_	Median (Years)	Range (Years)
Classic KS (23)	6.67 *	54.39	13.94	25.64	40.00	65.50	53.00 ^§^	32–73
AIDS-associated KS (5)	4.00 *	23.80	9.65	40.56	18.00	29.00	28.00 ^§^	10–34
Total (28)	6.00	48.93	17.74	36.25	37.00	63.00	50.50	10–73

M/F: male/female; SD: standard deviation; CV: coefficient of variation; P_25_: 25th percentile; P_75_: 75th percentile; * M/F ratios of two kind of KS has no significant difference (Fisher’s exact test); ^§^ *p* < 0.001 (ANOVA).

### 3.2. ORF-K1 Amplification

Total DNA were extracted from FFPET and plasmid samples. 363 bp of ORF-K1 VR1 region of KSHV were amplified by nested PCR from each sample. PCR products were detected by 1.5% agarose gel electrophoresis (see Supplementary File). DNA extracted from gel was cloned into pGEM-T vector, and then sent for sequencing.

### 3.3. Phylogenetic Analysis

**Figure 1:**
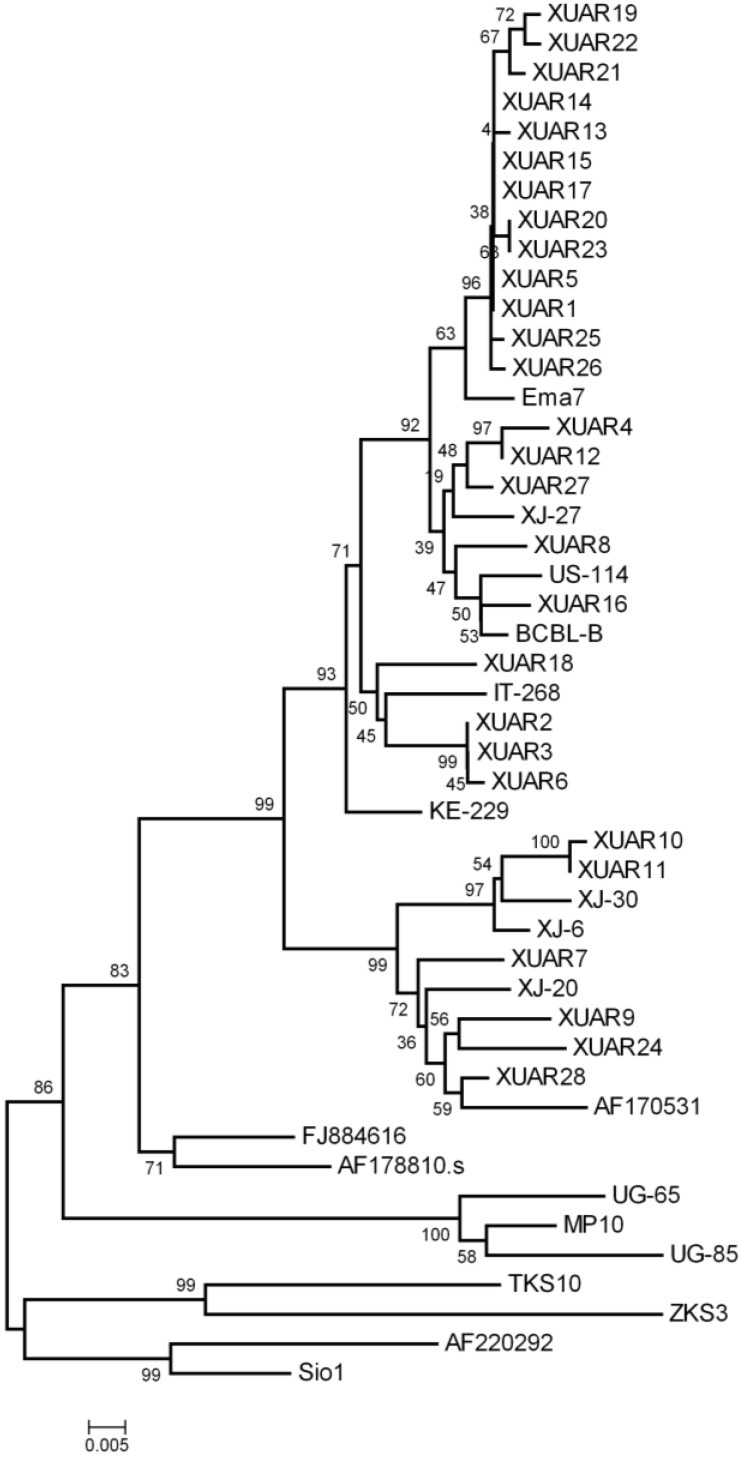
Phylogenetic tree of KSHV ORF-K1 DNA sequences constructed by MEGA (version 4.0.2) using the neighbor-joining algorithm. Relationships of KSHV isolates in present study and isolates in the literature are shown. KSHV isolates in present study were named by XUAR. XUAR1-XUAR23 were isolated from classic KS tissues, XUAR24-XUAR28 were isolated from AIDS-KS tissues. Ema7, US-114, BCBL-B, IT-268, KE229, XJ6, XJ20, XJ-27, XJ-30, AF170531, AF178810, AF220292, FJ884616, UG-65, UG-85, MP10, TKS10, ZKS3, and Sio1 were downloaded from NCBI.

The 363 bp K1 sequences of the 28 KSHV isolates from Xinjiang Uygur Autonomous Region, China were aligned with 19 previously reported sequences in the database of NCBI using Bioedit (version 7.0.0). Phylogenetic trees were constructed by neighbor-joining analysis by MEGA (version 4.0.2) and Phylip (version 3.68), respectively. From the MEGA phylogenetic trees (Figure 1), the Xinjiang cases were categorized into subtypes A and C. Twenty-three cases (82.14%) were categorized into subtype A and five cases (17.86%) categorized into subtype C in total. Of classic KS, 19 subjects (82.61%) were infected with subtype A and four subjects (13.91%) were infected with subtype C, while of AIDS-associated KS, four subjects (80.00%) were infected with subtype A and one subject (20.00%) was infected with subtype C. Subtype C2 and C3 and all known subtype A, except A5, were found in classic KS. In AIDS-KS, subtype A1, A2 and C3 were identified (Table 2). In this study, subtype A/C ratios in Classic KS and AIDS-associated KS were not significantly different (Fisher’s exact test); Subtype A/C ratios in Uygur patients and Kazakh patients were also not significant different (Fisher’s exact test, Table 3). By using neighbor-joining analysis with Phylip, we saw a similar topology (see Supplementary File).

**Table 2:** KSHV K1 genotypes between classic KS and AIDS KS.

KS Subtype	K1 Subtype *		
Classic KS			
	A (19)	A1	2
		A2	11
		A3	4
		A4	2
	C (4)	C2	2
		C3	2
AIDS-associated KS			
	A (4)	A1	1
		A2	3
	C (1)	C3	1

* KSHV subtype A/C ratios of classic and AIDS-KS were not significantly different (Fisher’s exact test).

**Table 3:** KSHV K1 genotypes in different ethnic patients.

KSHV Isolates	GenBank Accession Numbers	Ethnicity	Subtypes
XUAR1	KM582680	Uygur	A2
XUAR2	KM582681	Kazakh	A3
XUAR3	KM582682	Uygur	A3
XUAR4	KM582683	Uygur	A1
XUAR5	KM582684	Uygur	A2
XUAR 6	KM582685	Uygur	A3
XUAR7	KM582687	Uygur	C3
XUAR8	KM582688	Kazakh	A4
XUAR9	KM582689	Uygur	C3
XUAR10	KM582690	Uygur	C2
XUAR11	KM582691	Kazakh	C2
XUAR12	KM582692	Kazakh	A1
XUAR13	KM582693	Uygur	A2
XUAR14	KM582694	Hui	A2
XUAR15	KM582695	Uygur	A2
XUAR16	KM582696	Han	A4
XUAR17	KM582697	Uygur	A2
XUAR18	KM582698	Kazakh	A3
XUAR19	KM582699	Uygur	A2
XUAR20	KM582700	Kazakh	A2
XUAR21	KM582701	Kazakh	A2
XUAR22	KM582702	Uygur	A2
XUAR23	KM582703	Kazakh	A2
XUAR24	KM582704	Uygur	C3
XUAR25	KM582705	Uygur	A2
XUAR26	KM582706	Uygur	A2
XUAR27	KM582707	Uygur	A1
XUAR28	KM582708	Uygur	C3

KSHV subtype A/C ratios in different ethnic patients were not significantly different (Fisher’s exact test).

## 4. Discussion

Traditionally, herpesviruses are considered to be co-evolved with their hosts throughout vertebrate evolution. KSHV is an ancient human virus and spread together with its migrating human hosts [21]. Many studies have found seven subtypes of KSHV (A, B, C, D, E, F and Z) and identified that KSHV subtypes have close associations with the geographic and ethnic background of patients. Subtypes A and C have the largest prevalence area, including Africa, Europe, Middle East, Asia [12,22], America and Australia. Subtype D has been reported from the Pacific Islands, like Japan [12,16], Chinese Taiwan [23], and Australia. Subtype B is found from patients of African origin [24], only one subtype B isolates separated from a Mexican female AIDS patient in Miami [14]. Subtype E was only found in Amerindians [15,25]. Subtype Z has been found in Zambian [17]. Subtype F is rare and was only found in Africa recently [13].

In China, KS cases are rare, only reported in Xinjiang Region [20] and Taiwan Island [26]. However, KSHV subtypes in these regions are quite different from each other. Previous research and this study have found the KSHV subtypes in Xinjiang region are subtypes A and C, while in Taiwan it is subtypes C and D [23]. This difference may have geographic reasons. Taiwan is an island in the Pacific, while Xinjiang located in central Asia. Xinjiang is home to a number of different ethnic groups, including the Uyghur, Han, Kazakh, Hui, Kyrgyz, Xibo and Mongolians. Historically, the population in Xinjiang consisted of various nomadic tribes, like Yuezhi, Xiongnu, and Wusun. Well-preserved Tarim mummies with Caucasoid features, often with reddish or blond hair, which dated from 1800 BCE to 200 CE, which were different from the Han Chinese, have been found in Xinjiang area. Today the minorities, including Uyghur, Kazakh and Hui, still have significant difference from the Han. The epidemiology study has found that the seroprevalence of KSHV in the general population was higher than the other provinces in China, and the minorities (Uyghur, Kazakh, Hui, and Xibo) have higher KSHV-seropositive ratio than the Han [18]. Previous researches have suggested that KSHV has been introduced to Xinjiang Region along the Silk Road. The Silk Road was a path for cultural, commercial and technological exchange between Ancient China, India, the Persian Empire and Mediterranean countries for nearly 3000 years. The Silk Road was extended to the center of China, and Xinjiang was a section of the Silk Road. In our study, two subtypes of KSHV were identified, 23 of them belonged to subtype A, while five of them were subtype C. More genotype A was found than genotype C, both in Classical KS and AIDS KS. No significant difference was found in the predominance of different genotype between Classical KS and AIDS KS.

Co-migration of viruses with the human population is common. KSHV subtypes A and C are predominant in the Mediterranean, Middle Eastern and Asian regions [12,22], which were parts of the ancient Great Silk Road that went through the Xinjiang region. KSHV subtype C has been found in Xinjiang in 2001 [19]. KSHV subtype A has also been found in Xinjiang by our study in 2009 [18]. In this study, both KSHV subtype A and C were identified. Thus, it is tempting to speculate that KSHV might have been spread along the Silk Road with the human population.

Classic KS was only found in Xinjiang Minority, but not often seen in Han population. More epidemiologic study should be done to investigate the risk factors, including environmental and human genetic factors.

## Supplementary Files

Supplementary File 1
