# Supplementary material for: Genotypic Analysis of Kaposi’s Sarcoma-Associated Herpesvirus from Patients with Kaposi’s Sarcoma in Xinjiang, China
Source: Viruses. 2014 Nov 26;6(12):4800–10. doi: 10.3390/v6124800 (PMC4276930; doi:10.3390/v6124800)
Supplement: Supplementary File 1 [file viruses-06-04800-s001.pdf]

Supplementary Materials

Genotypic Analysis of Kaposi's Sarcoma-Associated Herpesvirus from Patients with Kaposi's Sarcoma in Xinjiang, China

Xinxing Ouyang, Yan Zeng, Bishi Fu, Xiaowu Wang, Wei Chen, Yuan Fang, Minhua Luo and Linding Wang

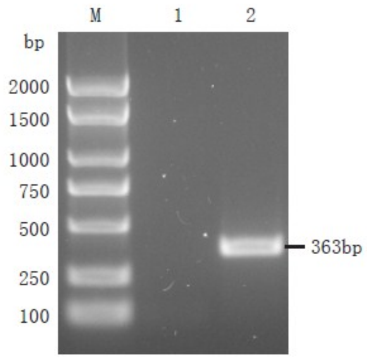

Figure S1. 363 bp of KSHV K1 gene amplified by nested-PCR. M, mark; 1, negative control; 2, Nested-PCR product of DNA sample extract from KS tissue.

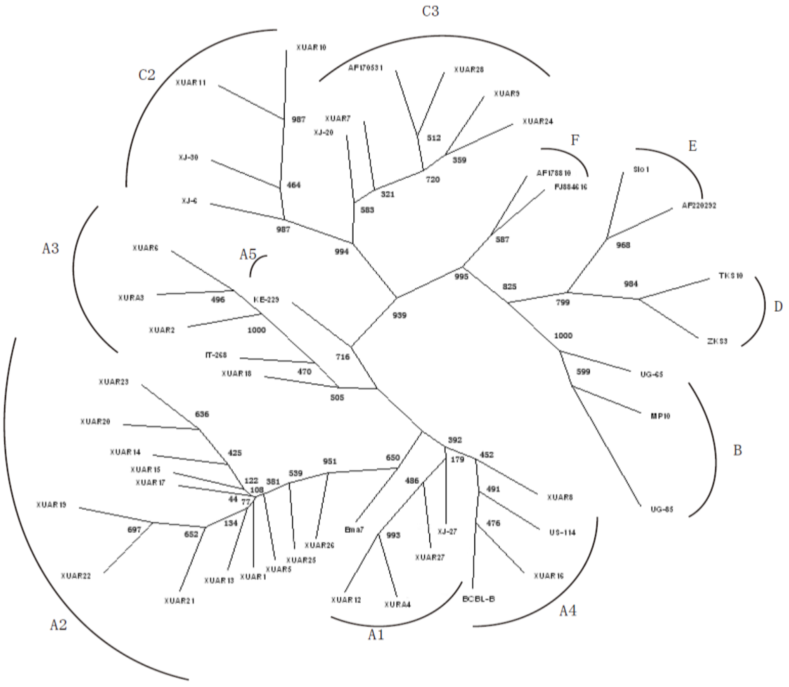

Figure S2. Phylogenetic tree of KSHV ORF-K1 DNA sequences constructed by phylip (version 3.68) using the neighbor-joining algorithm (1000 replicates). Relationships of KSHV isolates in present study and isolates in the literature were shown. KSHV isolates in present study were named by XURA. XUAR1-XUAR23 were isolated from classic KS tissues, XURA24-XUAR28 were isolated from AIDS KS tissues. Ema7, US-114, BCBL-B, IT-268, KE229, XJ6, XJ20, XJ-27, XJ-30, AF170531, AF178810, AF220292, FJ884616, UG-65, UG-85, MP10, TKS10, ZKS3, Sio1 were download from NCBI.
